# Graft-to-Footprint Mismatch Is Not Associated with Early ACL Reconstruction Failure: A Retrospective Cohort Study

**DOI:** 10.3390/sports14090367

**Published:** 2026-08-25

**Authors:** Alex Quok An Teo, Zavier Yongxuan Lim, Janice Hui En Tan, Joel Zhao Jie Lee, Hoi Pong Nicholas Wong, Fucai Han

**Affiliations:** 1 Department of Orthopaedic Surgery, Ng Teng Fong General Hospital, Singapore 609606, Singapore; alex_teo@nuhs.edu.sg (A.Q.A.T.);; 2 Department of Orthopaedic Surgery, National University Hospital, Singapore 119074, Singapore

**Keywords:** knee, anterior cruciate ligament, graft, reconstruction

## Abstract

**Purpose**: In a relatively new concept of individualised ACL reconstruction, the restoration or matching of the native ACL footprints at both the tibial and femoral insertions might be sufficient as a surgical objective. This study aimed to determine whether ACL graft size relative to the native ACL tibial footprint influences the rate of graft failure in a Singaporean Asian population. **Methods**: A retrospective study of all consecutive patients who underwent primary hamstring autograft ACL reconstruction over a 3-year period at a tertiary referral centre was performed. Baseline anthropometric data and native ACL tibial footprint area from pre-operative MRI scans were collected. Graft area was calculated based on the graft tunnel diameter. ACL graft failures were diagnosed clinically and confirmed radiologically. Multivariable analyses were performed using pre-specified variables based on the literature. **Results**: This cohort comprised 240 patients with a mean age of 24.4 years. There were a total of seven graft failures (2.92%), with two patients undergoing revision surgeries. There was no association between graft-to-footprint ratios and failure rates (71.9 vs. 80.2%, *p* = 0.386). There were no other significant predictors of graft failure. There was no statistically significant difference in graft failure rate between grafts <8 mm and ≥8 mm (*p* = 1.00). Gender, but not the graft-to-footprint ratio, was a significant predictor of graft size. **Conclusions**: No statistically significant association was identified between graft-to-footprint ratio and early graft failure. However, the low event rate and short-term follow-up limit definitive conclusions.

## 1. Introduction

Anterior cruciate ligament (ACL) tears remain a common orthopaedic injury worldwide, with some reports indicating increasing rates over the past two decades [1,2]. As we continue to uncover the protective benefits of ACL reconstruction on the knee in preventing secondary chondral and meniscal injuries [3], rates of reconstruction have also concordantly risen over the same period. An epidemiological analysis performed in Australia from 2000 to 2015 showed a 43% increase in annual incidence of ACL reconstructions over the period and a significant 74% increase for patients under 25 years old [4].

ACL graft failures are thought to occur at a rate of about 4.6% [5] but vary widely up to as high as 34% at 15 years [6], depending on various patient and surgery-related factors. Graft size has been reported to be one of the major factors affecting failure rates, with larger graft sizes generally being preferred to minimise the risk of failure. A recent meta-analysis reported significant decreases in the hamstring autograft failure rate, corresponding with 0.5 mm increments in graft diameter from <7 mm to >9 mm [7]. This relationship, however, is not universally true, with some studies reporting that smaller grafts do not lead to more failures [8,9]. Grafts that are too large risk being impinged within the femoral notch during knee movement, which has been theorised to affect graft healing [10] and even the functioning of the native posterior cruciate ligament (PCL) [11].

The concept of individualised ACL reconstruction has been introduced in an attempt to optimise patient outcomes [12]. It emphasises recognition of patients’ unique circumstances, including their anatomy both in terms of tunnel placement and native ACL footprint area, and the selection of grafts [13] to restore those appropriately [14]. The restoration or filling of the native ACL footprints at both the tibial and femoral insertions is often touted as a surgical objective. The correlation of this with clinical outcomes, however, has not been fully established.

The aim of this study was to determine whether ACL graft size relative to the native ACL tibial footprint influences the rate of ACL graft failure in a smaller Asian population. We hypothesised that the closer the ACL graft size relative to the native footprint, the lower the failure rate.

## 2. Materials and Methods

**Figure 1:**
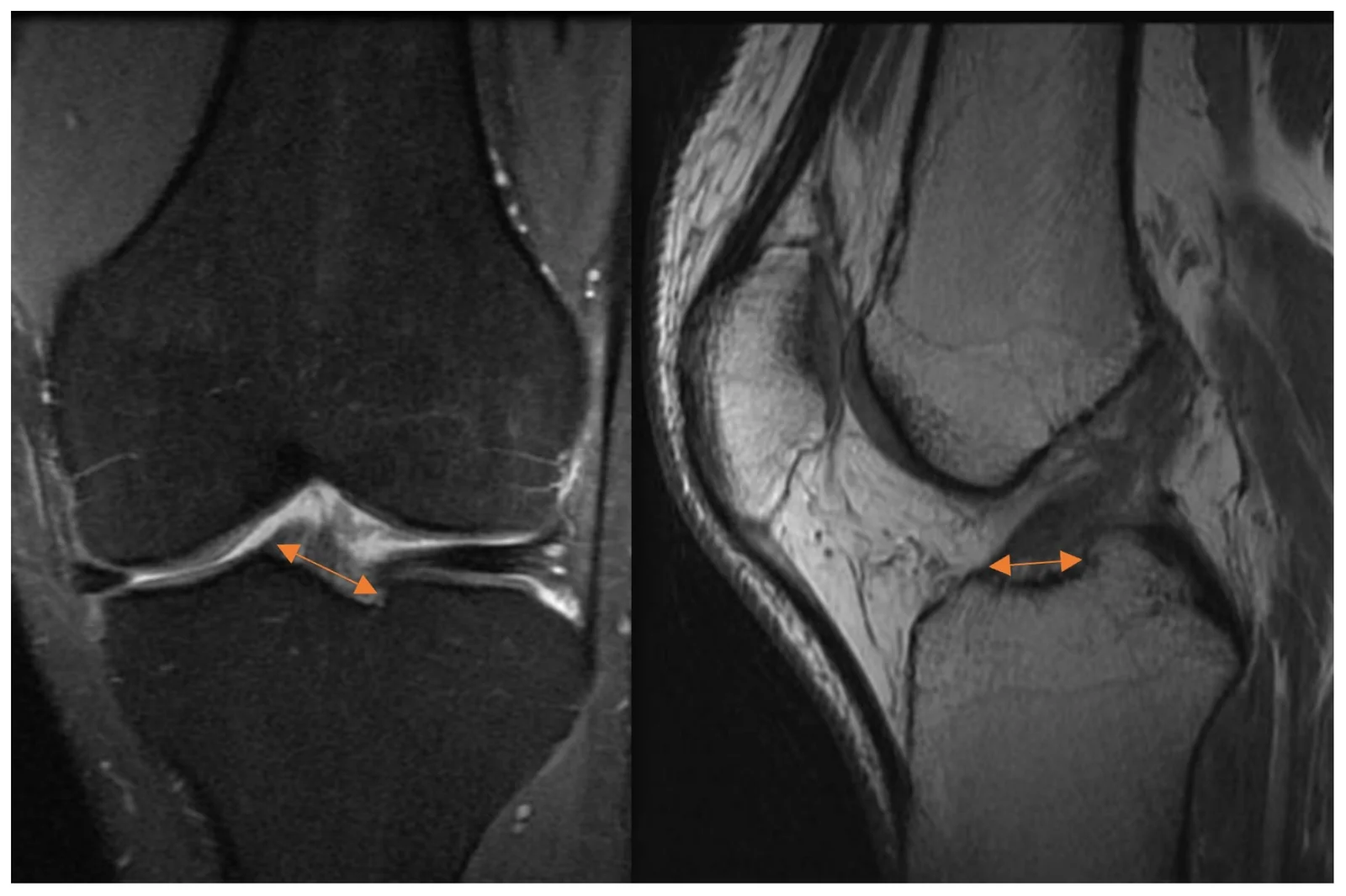
Estimation of native ACL footprint anteroposterior and mediolateral lengths based on coronal and sagittal cuts on pre-operative magnetic resonance imaging (MRI) scans.

We performed a retrospective cohort study of all consecutive patients who underwent primary ACL reconstruction with a hamstring autograft over a 3-year period from January 2016 to December 2018 at a tertiary referral centre. Ethical approval was obtained from the local institutional review board prior to commencement, and the need for informed consent was waived due to its retrospective nature. All reconstructions were performed by two surgeons who underwent specific sports surgery fellowships. There was a median follow-up duration of 11.8 months. We collected baseline patient anthropometric data, and the native ACL tibial footprint area was approximated as an ellipse using pre-operative MRI-derived anteroposterior and mediolateral lengths on sagittal and coronal cuts (Figure 1). Both measurements were chosen based on the most anterior to most posterior fibres of the ACL or the most medial to most lateral fibres, respectively, parallel to the tibial plateau. The formula used to estimate the tibial footprint was anteroposterior length × mediolateral length × π/4. Measurements were performed using a standardised protocol by two independent observers who were blinded to the treatment outcome. Any discrepancy in measurement values of more than 10% was resolved by consensus discussion, and any unresolved disagreements were resolved by an independent third party. Graft area was calculated based on the graft tunnel diameter using the formula π × (graft tunnel diameter/2)^2^. The graft-to-tibial footprint ratio was calculated as (graft area/tibial footprint area) × 100%. This study focused on tibial footprint measurement only as it is more reliably visualised and measurable on MRI scans. For this purpose, we assumed homogeneity of the diameter of the native ACL graft and that matching of the tibial footprint area would result in matching of the femoral footprint area.

Graft fixation was performed with either a suspensory device or an interference screw on either end according to surgeon preference. Supplementary fixation with a tibial post screw was employed when deemed necessary. ACL graft failures were diagnosed clinically and confirmed on MRI during the period of follow-up. Clinical failure of the reconstruction was defined by patient-reported symptoms of persistent or recurrent instability, coupled with a grade 1 pivot shift over two consecutive visits or a grade 2 pivot shift at any time post-operatively.

### Statistical Analysis

Shapiro–Wilk tests were performed for continuous variables, and descriptive statistics including mean and standard deviation or median and interquartile range were reported for normally distributed and non-parametric data, respectively. Univariate analyses with T-tests, Mann–Whitney U tests and Fisher’s exact tests were performed. Graft failure was modelled using Firth’s penalised logistic regression via the logistf package in RStudio (version 2026.08.0). Multivariate linear regression analyses were then performed, with a *p*-value < 0.05 being statistically significant. As each linear regression model addressed separate, pre-defined outcomes, no adjustment for multiple comparisons was applied. In each regression model, no stepwise selection procedures were applied. There was no missing data for variables included in regression analyses. All other statistical analyses were performed on IBM SPSS 28 Software (IBM Corp., Armonk, NY, USA).

## 3. Results

A total of 240 patients were included in this study. The baseline demographics of the patients in our study are shown below in Table 1. There was an equal number of female and male patients in our study.

Only seven graft failures (2.92%) were reported during the period of follow-up, limiting statistical power for detecting associations. Two of the patients with graft failures underwent revision surgeries. One of the seven patients sustained a clinical failure and declined MRI. He did not undergo revision. We performed a univariate comparison of baseline and clinical variables between patients with and without graft failure (Table 2). We performed Firth’s penalised logistic regression to determine any predictive or protective factors for graft failure but did not find any among the variables studied (Table 3).

We also examined the influence of graft size on the rate of graft failure. There was no statistically significant difference between grafts <8 mm and ≥8 mm in the graft failure rate (*p* = 1.00), as shown in Table 4 below.

A multivariate linear regression analysis was performed to examine factors affecting the eventual graft size utilised in reconstruction. Of these, gender was the only significant variable for graft size (*p* = 0.020), as shown in Table 5 below.

We also investigated if anthropometric factors were able to predict tibial footprint area, as shown in Table 6 below. Height was the only borderline predictor of tibial footprint area among the variables studied (*p* = 0.050).

## 4. Discussion

This study revealed that graft size and footprint coverage matching are unlikely to be dominant determinants of early graft failure, with the caveat of a very low number of failure events, which substantially limits statistical power and increases the risk of type II error.

The increasing popularity of ACL reconstructions in recent years has placed increased scrutiny on graft failure rates in an attempt to optimise patient outcomes. There is ongoing debate in the current literature about the various factors thought to influence graft failure. A recent systematic review of 28 studies [15] found recurrent trauma to be the single most important cause of failure but also recognised that, in a significant proportion of failures, multiple factors may be implicated. The literature surrounding the influence of graft size on failure rates has been mixed over the past decade. Larger graft diameters are generally recommended as they are thought to be able to better withstand any future stresses; however, even large registry studies have failed to find a consistent association between graft size and failure rate. A study of 992 patients from the New Zealand ACL registry [16] and 4029 patients from the Norwegian Knee Ligament Registry found no association between graft sizes and a higher rate of revision surgeries [9]. Further, Boots et al. [8] found that even reconstructions with smaller graft diameters allowed an equivalent rate of return to sports in smaller female athletes. These conflicting results again hint at the multifactorial nature of graft failures and that the need for larger grafts may not universally apply. Inappropriately large ACL grafts may instead risk complications, including impingement or tunnel blowout.

The concept of individualised anatomic ACL reconstruction may partially explain this inconsistent association between graft size and failure. This principle emphasises a more thorough evaluation of a patient’s individual anatomical features in order to be able to reconstruct it more accurately. It has been suggested that, among other things, the reconstructed ACL graft size should restore the native ACL footprint [17]—both in size and location—in order to achieve a satisfactory outcome [18]. This would help guide not only graft size but also choice of reconstruction technique (e.g., single- vs. double-bundle) and drill angles [17]. It may simply be that in patients with smaller native footprints, large graft sizes may not be necessary and furthermore that those that are too large may paradoxically increase the rates of failure due to the abovementioned impingement affecting graft incorporation. Our study did not show a statistically significant difference in the graft-to-tibial native footprint coverage ratio in patients with graft failures as compared to patients without graft failures. Sasaki et al. [19] found in their cadaveric study that knee biomechanics were poorer than the intact knee in single- or double-bundle reconstructions that covered 50% or less of the native femoral ACL footprint. Similarly, a finite element analysis study by Yang et al. [20] found that better coverage of the femoral ACL footprint reduced graft stress. Another finite element analysis study found that matching the graft to the native ACL size—measured as an average of the insertion sites and the ligament isthmus—more effectively stabilised the reconstructed knee [21].

A possible explanation for this apparent lack of association could be the relatively short study follow-up period. Webster and Feller previously reported that the majority of graft ruptures in their cohort occurred within the first two years post-operatively, with 47% occurring within the first year [22]. This is believed to be partly due to the ligamentisation of the graft that occurs during this period [23], with early return to sport also identified as a significant risk factor [24]. A more recent meta-analysis by Khan et al. [25], however, found that the mean time to graft rupture for a pooled 2974 patients was 41.1 months. It is thus conceivable that the graft-to-footprint matching—or lack thereof—plays a more important role in the longer-term biomechanical performance of the graft. Longitudinal follow-up over a longer period in larger cohorts would be illuminating in this regard.

The recent attention paid to recognition of the unique nature of any given patient’s anatomy extends also to attempts at predicting the sizes of the harvested autografts. Pre-emptively identifying cases where the harvested autografts may be insufficient to provide a graft of ‘adequate’ diameter would allow alternative plans to be made for choice of graft. Again, in this regard, the literature over the past two decades has been relatively mixed with regard to being able to pre-operatively predict graft sizes from patient anthropometric data. A previous systematic review found patient height to be the most consistent predictor of graft size [26]. A more recent meta-analysis of 42 studies involving 7110 ACL reconstruction patients reported a correlation between graft size and patients’ height, weight, thigh circumference, thigh length and BMI [27]. These relationships held true with subgroup analysis of hamstring autografts alone, which made up the bulk of the studies. Our study found that, of all the anthropometric factors, only gender (*p* = 0.020) was significantly correlated with graft size, with female patients having smaller harvested hamstring autografts in general compared to males. This is consistent with many of the previous studies in the literature which have studied this association [28,29,30,31].

Despite this, in our population, only height demonstrated a borderline association with native tibial footprint area (*p* = 0.05), not reaching the pre-specified significance threshold. There were no significant differences between genders. A pilot study on 53 Indian subjects did not find any significant differences in ACL tibial footprint length and width with patient height, weight or gender of patients [32]. Another study on 70 Indian patients reported no differences in average ACL tibial insertion size between genders [33].

This study is not without its limitations, which include the overall small number of ACL graft failures in our cohort, which is at least partly related to the short mean duration of follow-up. This, with the inability to detect a significant difference with native footprint coverage, may reflect an underpowered study. Estimation of the native ACL tibial footprint from the pre-operative MRI measurement may not truly reflect the actual footprint area, but we believe the consistency of the measurement method across the cohort should provide an acceptable gauge of the footprint area. Direct intra-operative measurement of the footprint areas in a larger cohort of patients should be a focus for future work. Additionally, graft size was estimated indirectly from tibial tunnel diameter. Direct calliper-based graft measurements may provide more precise alternatives for future studies. There was also the factor of subjectivity in surgeon decision-making, with two surgeons in this cohort, thus introducing heterogeneity. We were also unable to control for other factors that may have impacted graft failure rates, including the timing of return to activity and tunnel placement. The use of multivariable modelling with a low number of events increases the risk of overfitting and limits the reliability of these estimates.

A key limitation is the short duration of follow-up (median 11.8 months, IQR 6.9–15.8) relative to the reported natural history of graft rupture. Khan et al. reported a pooled mean time to graft rupture of 41.1 months, albeit with wide variation across cohorts (range 8.0–82 months) [25]. Using a constant-hazard approximation anchored to this mean, only ~26% of eventual failures would be expected to have manifested within our cohort’s mean follow-up of 12.6 months, implying an expected total of approximately 26–27 failures rather than the seven observed. A similar estimate (~26 expected failures) is obtained independently, applying Khan et al.’s pooled overall rupture rate of 11% to our cohort size. These approximations should be interpreted as illustrative rather than precise, given their dependence on an assumed hazard shape, but they suggest that our observed failure rate likely substantially underestimates the true rate and the null association between graft-to-footprint ratio and failure should be interpreted accordingly.

## 5. Conclusions

In this clinical study assessing the clinical impact of native ACL footprint coverage on failure and revision rates post-ACL reconstruction surgery, we did not find any detectable association in the short-term. It is plausible that graft-to-footprint mismatch influences longer-term graft integrity or biomechanical performance rather than early clinical failure, which is more commonly driven by technical factors or reinjury. As we continue to strive towards anatomical individualised ACL reconstructions as the gold standard, future work should focus on assessing this relationship across larger cohorts while controlling for other potential confounding factors, with a longer duration of follow-up. This would help us define the various vital aspects of the individualised reconstruction in order to achieve optimal patient outcomes.

## Figures and Tables

**Table 1:** Baseline demographics of the patient cohort.

Baseline Demographics	N/Mean ± S.D./Median (IQR)
Gender	
Male	120
Female	120
Age	24.4 ± 6.07
Height/cm	170.78 ± 6.98
Weight/kg	72.1 (62.63, 82.73)
Body Mass Index (BMI)/kg m^−2^	24.4 (21.6, 28.5)
Operation Side	
Left	120
Right	120
Graft Size/mm	8.0 (7.5, 8.5)
<8 mm	90
≥8 mm	150
Number of Strands	
3	2
4	191
5	17
6	9
7	1
8	2
Data unavailable	18
Tibial tunnel diameter/mm	8.00 ± 0.720
Femoral tunnel diameter/mm	8.00 ± 0.706
Tibial footprint area/mm^2^	65.04 (55.42, 75.82)
Mean follow-up duration	12.6 months
Median follow-up duration	11.8 months (6.9, 15.8)

**Table 2:** Univariate comparison of baseline and clinical variables between patients with and without graft failure. M, male; F, female. L, left; R, right.

	Graft Failure	No Graft Failure	*p*-Value
Age [years]	24.29 ± 7.70	24.39 ± 6.04	0.966
Gender	4 M/3 F	116 M/117 F	1.000
Height [cm]	169.43 ± 9.32	170.82 ± 6.88	0.605
Weight [kg]	72.00 (62.70, 82.75)	79.00 (59.70, 83.10)	0.965
BMI [kg/m^2^]	26.12 (21.02, 27.38)	24.40 (21.60, 28.54)	0.869
Operation Side	4 L/3 R	116 L/117 R	1.000
Graft:Tibial footprint Ratio/%	71.9 ± 23.4	80.2 ± 25.0	0.386

**Table 3:** Firth’s penalised logistic regression for graft failure. Likelihood ratio test = 5.64, df = 7, *p* = 0.582.

	B	SE	*p*-Value	Odds Ratio	95% CI
Age [years]	0.036	0.055	0.591	1.04	[0.90, 1.17]
Female	1.756	0.926	0.098	5.79	[0.71, 48.16]
Height [cm]	−0.204	0.191	0.363	0.82	[0.52, 1.28]
Weight [kg]	0.272	0.208	0.269	1.31	[0.80, 2.18]
BMI [kg/m^2^]	−0.749	0.619	0.298	0.47	[0.10, 1.99]
Operation Side	−0.124	0.649	0.864	0.88	[0.19, 3.76]
Graft:Tibial footprint Ratio/%	−1.752	1.478	0.295	0.17	[0.00, 3.76]

**Table 4:** Fisher’s exact test for graft size and graft failure (*p* = 1.00).

	<8 mm	≥8 mm
Graft failure	3	4
No Graft Failure	87	146

**Table 5:** Multivariate linear regression for anthropometric factors on graft size. F = 10.618, *p* < 0.01, R^2^ = 0.133, adjusted R^2^ = 0.118.

	B	SE	t	*p*-Value	95% CI
Gender	−0.301	0.129	−2.334	0.020	[−0.555, −0.047]
Height [cm]	−0.028	0.032	−0.880	0.380	[−0.092, 0.035]
Weight [kg]	0.056	0.036	1.533	0.127	[−0.016, 0.127]
BMI [kg/m^2^]	−0.139	0.107	−1.302	0.194	[−0.350, 0.071]

**Table 6:** Multivariate linear regression for anthropometric factors on tibial footprint area. F = 6.520, *p* < 0.01, R^2^ = 0.100, adjusted R^2^ = 0.085.

	B	SE	t	*p*-Value	95% CI
Gender	−5.494	3.778	−1.454	0.147	[−12.938, 1.950]
Height [cm]	1.807	0.918	1.968	0.050	[−0.002, 3.616]
Weight [kg]	−1.369	1.034	−1.324	0.187	[−3.406, 0.668]
BMI [kg/m^2^]	3.895	3.037	1.282	0.201	[−2.089, 9.879]

## Data Availability

The dataset supporting the conclusions of this article is available upon request to the corresponding author.
